# Immune thrombotic thrombocytopenic purpura and diabetic ketoacidosis: a case report and literature review

**DOI:** 10.1186/s12959-025-00740-w

**Published:** 2025-05-19

**Authors:** Geng-Hao Bai, Mei-Hwa Lin, Yu-Pei Chen, Tien-Jyun Chang, Sheng-Chieh Chou

**Affiliations:** 1 Department of Internal Medicine, National Taiwan University Hospital, Taipei City, Taiwan; 2 Department of Laboratory Medicine, National Taiwan University Hospital, Taipei City, Taiwan; 3 Division of Metabolism and Endocrinology, Department of Internal Medicine, National Taiwan University Hospital, Taipei City, Taiwan; 4 Division of Hematology, Department of Internal Medicine, National Taiwan University Hospital, Taipei City, Taiwan

**Keywords:** Thrombotic thrombocytopenia purpura, Diabetic ketoacidosis, Acute kidney injury

## Abstract

**Background:**

Thrombotic thrombocytopenic purpura (TTP) is an uncommon and life-threatening disorder caused by a deficiency of ADAMTS-13, and eventually leads to microangiopathic hemolytic anemia, severe thrombocytopenia, and organ damages. Acute TTP events could be triggered by infections, or inflammations in the context of ADAMTS-13 deficiency. Recently, several case reports have indicated an association between diabetic ketoacidosis (DKA) and TTP. Here, we present a case with the concomitant presentation of DKA and TTP.

**Case presentation:**

A 37-year-old male with diabetes mellitus presented with typical symptoms of diabetic ketoacidosis. He was managed with an insulin pump and intravenous fluids. However, he developed seizure and progressed to coma, his rapidly deteriorating condition necessitated continuous renal replacement therapy, intubation, and inotropic support. Laboratory data indicated hemolytic anemia and thrombocytopenia, and a blood smear revealed schistocytes. The PLASMIC score was 5, and ADAMTS-13 activity was 2%. The patient was diagnosed with TTP and treated with therapeutic plasma exchange, steroids, and rituximab. His platelet count stabilized above 150,000/µL, and ADAMTS-13 activity progressively improved.

**Conclusions:**

This case report emphasizes the concurrence of DKA and iTTP, presenting the rare complication of acute renal failure in TTP. TTP is a rare and serious disease that requires prompt recognition and management. Concurrent conditions should be considered when calculating prediction scores such as the PLASMIC and French scores.

**Supplementary Information:**

The online version contains supplementary material available at 10.1186/s12959-025-00740-w.

## Background

Thrombotic thrombocytopenic purpura (TTP) is a rare and life-threatening thrombotic microangiopathy characterized by microangiopathic hemolytic anemia, severe thrombocytopenia, fever, neurological symptoms, and kidney injury [1]. TTP is caused by a severe deficiency of the von Willebrand factor (vWF) cleaving protease, ADAMTS-13 (a disintegrin and metalloprotease with thrombospondin type 1 repeats, member 13) [2–5]. Most ADAMTS-13 deficiencies are due to autoantibodies, a condition known as immune TTP (iTTP). iTTP requires rapid diagnosis and urgent management, including therapeutic plasma exchange (TPE), and immunosuppressants such as steroids and rituximab [1, 2, 4, 6, 7]. The development of autoantibodies against ADAMTS-13 is generally idiopathic [1, 5, 8, 9], but acute TTP episodes could be triggered by infections, inflammations or pregnancy. On the other hand, diabetic ketoacidosis (DKA) is one of the most common life-threatening complications of diabetes mellitus. While it predominantly affects patients with type 1 diabetes, those with type 2 diabetes are also susceptible, particularly during periods of acute illness and catabolic stress. Appropriate treatment has reduced the mortality of DKA to less than 1%; however, the metabolic decompensation precipitated by underlying medical illness remains a leading cause of death in patients with DKA [10]. Several case reports have shown that TTP or similar thrombotic microangiopathy can develop concomitantly with DKA [11–21]. Here, we present a case of a man with type 2 diabetes mellitus who developed iTTP during an episode of DKA and reviewed all similar case reports from the literature.

## Case presentation

A 37-year-old male without medical history, presented with mild generalized abdominal pain, nausea, vomiting, watery diarrhea, and oliguria for two days. Initially, he sought medical assistance at a local clinic, where acute viral gastroenteritis was suspected, and he was treated with hydration therapy. However, his symptoms worsened the following day, prompting a visit to the emergency department of a local hospital. Upon arrival, he experienced a generalized seizure lasting two minutes during triage. Hypertension (172/108 mmHg), icteric sclerae, and petechiae on the limbs and trunk were noted. Initial laboratory findings indicated extreme hyperglycemia (721.8 mg/dL), high anion gap metabolic acidosis (pH 7.2, bicarbonate 12.2 mmol/L, anion gap 20.8 mEq/L), hyperkalemia (5.1 mmol/L), and elevated blood ketones with an HbA1c of 11.5%. Additional findings included anemia (hemoglobin 10.8 g/dL), thrombocytopenia (16 × 10^9^/L), indirect hyperbilirubinemia (total bilirubin 3.40 mg/dL, direct bilirubin 0.94 mg/dL), mildly elevated aspartate transaminase (61.5 U/L), high blood urea nitrogen (91.29 mg/dL) and creatinine (8.53 mg/dL). Coagulation screening tests, including prothrombin time, partial thromboplastin time, and fibrinogen were normal.

**Fig. 1:**
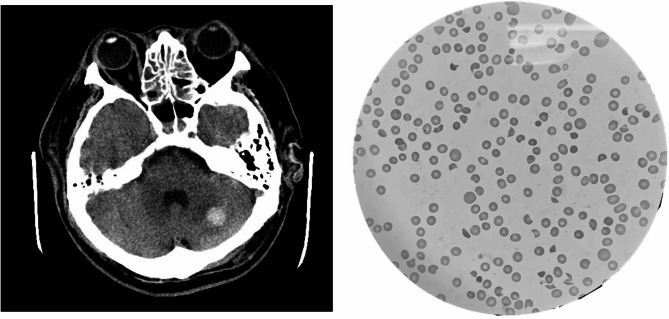
(**a**) Brain computed tomography of left cerebellum intracerebral hemorrhage; (**b**) Peripheral blood smear of schistocyte

The patient was admitted to the intensive care unit (ICU) on the same day and was managed with an insulin pump while continuous renal replacement therapy (CRRT) was initiated. A transfusion of platelets was administered; however, the patient’s condition worsened the following day, characterized by disturbed consciousness, hypoxia, and shock. Norepinephrine was administered and he was intubated with ventilator support. Whole-body computed tomography (CT) revealed only left cerebellum hemorrhage (Fig. 1a). After five days of management in the local hospital, the patient was transferred to a tertiary care center ICU for further evaluation of the unidentified cause of thrombocytopenia and unstable hemodynamics.

**Fig. 2:**
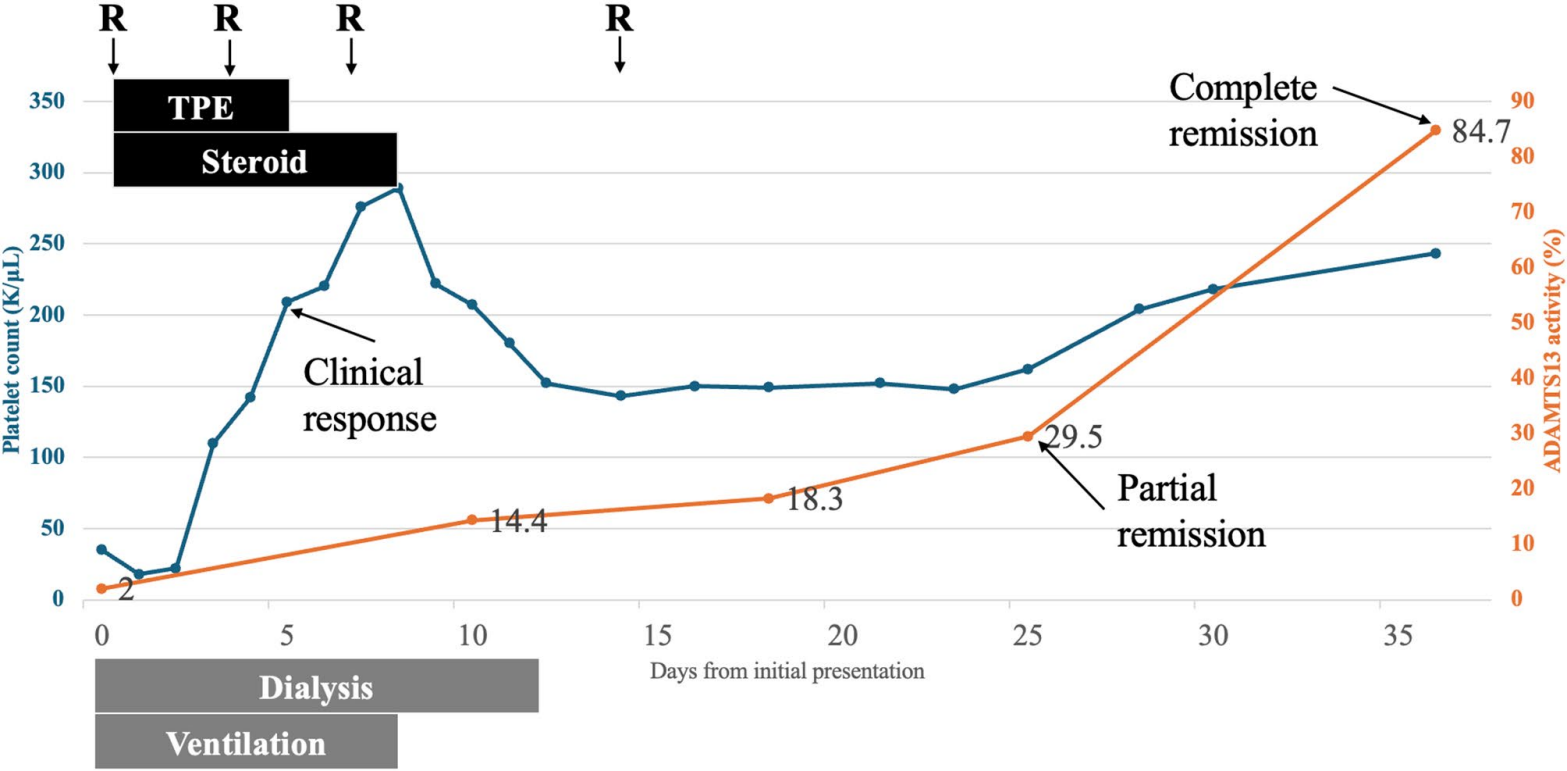
Platelet counts and ADAMTS-13 activity. The blue curve represents the platelet count and corresponds to the left axis, while the orange curve represents ADAMTS-13 activity and corresponds to the right axis

On arrival, patient was in a complete coma (E1VTM1) with worsening hemolytic anemia and thrombocytopenia (12 × 10^9^/L). Both direct and indirect Coombs tests were negative. An initial blood smear revealed multiple schistocytes (Fig. 1b). The PLASMIC score was 5 and the French score was 1, suggesting an intermediate risk of TTP (Table 1). It took one day to receive the ADAMTS-13 activity result, which was 2%, confirming a severe deficiency of ADAMTS-13 and TTP. Therapeutic plasma exchange, initiated on the day the ADAMTS-13 result was confirmed and was performed for five consecutive days until platelet count rose above 150 × 10^9^/L. Steroid and rituximab were also applied at the initiation of TPE. Specifically, methylprednisolone was given 80 mg daily until the cessation of TPE and then tapered over three days. Rituximab (fixed dose, 500 mg) was administered on day 1, 4, 7, and 14. ADAMTS-13 activity was monitored weekly after clinical remission, achieving ADAMTS-13 partial remission at day 25 (29.5%), and complete remission at day 36 (84%) (Fig. 2); moreover, genetic testing for ADAMTS13 revealed no mutations.

**Table 1:** PLASMIC score and French score of the patient

Parameter	Laboratory data	PLASMIC score [22]	French score [23]
Platelet count	12 × 10^9^/L (< 30 × 10^9^/L)	1	1
Creatinine level	5.63 mg/dL (< 2 mg/dL; ≤2.26 mg/dL)	0	0
Parameters of hemolysis	Reticulocytosis, indirect bilirubinemia, highly elevated LDH, undetectable haptoglobin.	1	
Associated conditions	No active cancer, No history of solid-organ or hematopoietic stem cell transplant	1	
MCV	90.4 fL (< 90 fL)	0	
INR	1.01 (< 1.5)	1	
ANA	1:80 (positive ANA)		0
Total score (Risk category)	PLASMIC score: 5 (Intermediate risk, risk of TTP: 5 ~ 24%) [20] French score: 1 (Intermediate risk, risk of TTP: 70%) [11]		

Continuous renal replacement therapy was transitioned to hemodialysis on the first day of plasma exchange. The patient’s consciousness improved following the initiation of therapeutic plasma exchange. We monitored blood glucose levels, blood gases, anion gap, and serum ketones in accordance with the DKA management protocol. The DKA showed significant improvement within two days of initiating continuous insulin infusion. Glycemic control improved further following the tapering of steroid therapy (Supplemental Fig. 1). The patient’s oxygen saturation and urine output showed gradual improvement. He successfully passed the spontaneous breathing trial and weaning parameters on day seven, leading to successful extubation on day eight. He was subsequently transferred to the general ward on day nine, and hemodialysis was paused on day ten. Blood urea nitrogen (BUN) and creatinine levels improved, negating the need for further hemodialysis. A glucagon test confirmed preserved pancreatic beta-cell function, facilitating the transition from insulin to oral antidiabetic medication once blood sugar levels were controlled. Kidney function remained stable over time. Prior to discharge, he regained the ability to care for himself independently, perform calculations accurately, walk long distances, and climb stairs without assistance.

## Discussion

Accurate diagnosis of immune thrombotic thrombocytopenic purpura (iTTP) necessitates clinical awareness. As demonstrated by this case, iTTP was not considered until the patient was transferred to a tertiary medical center. The presence of diabetic ketoacidosis (DKA) complicated the clinical picture, rendering the presentation of TTP atypical. Moreover, the primary physicians initially focused solely on managing the DKA and its complications, overlooking signs such as hemolytic anemia and thrombocytopenia that warranted further investigation. It is imperative to enhance the awareness of intensivists and other physicians managing critical care settings to facilitate the accurate diagnosis and treatment of iTTP, particularly in complex cases such as this one.

The PLASMIC score [22] and the French score [23] are both useful tools for identifying patients with severe ADAMTS-13 deficiency before ADAMTS-13 results are available. However, in our patient, both scores yielded intermediate results, with a predicted risk of TTP ranging from 5 to 24% using the PLASMIC score and 70% using the French score [24]. Lee et al. [25, 26] further validated the predictive accuracy of the PLASMIC score in Taiwanese population, finding that MCV did not significantly contribute to risk stratification. Tang et al. also demonstrated that MCV < 90 fL was not an independent predictor of TTP. A simpler scoring system that omits MCV < 90 fL may be more suitable for identifying TTP in Chinese patients [27]. These findings suggest that MCV < 90 fL may be less relevant in predicting TTP among East-Asian populations, as evidenced by this patient’s initial MCV of 90.4 fL. Furthermore, both prediction models indicate that kidney injury is less commonly observed in TTP compared to atypical hemolytic uremic syndrome (aHUS). In this report, the patient had complicated with DKA and severe kidney injury, which could also hamper the accuracy of both scores. Most renal dysfunction in TTP is mild, but acute renal failure requiring dialysis still occurs in 4–15% of TTP patients [28]. Generally, severe renal injury happens more frequently among patients with atypical hemolytic uremic syndrome (aHUS) than those with iTTP [29]. However, a retrospective study of 92 cases of TTP with low ADAMTS-13 activity (< 10%) found that more than half of the patients had acute kidney injury, with nearly half experiencing stage 3 acute kidney injury and a quarter requiring renal replacement therapy [30]. This case highlights the relatively rare presentation of iTTP. In retrospect, uncontrolled diabetes mellitus, hypertension, and subsequent DKA may have contributed to the kidney injury more than iTTP itself in this case. Similarly complicated situation might happen in other iTTP patients, therefore extra caution should be taken while interpretating kidney injuries in possible iTTP patients.

Caplacizumab is a humanized immunoglobulin fragment that targets the A1 domain of von Willebrand factor (vWF), thereby inhibiting its interaction with the platelet glycoprotein Ib-IX-V receptor. Phase 2 and 3 clinical trials have demonstrated that caplacizumab treatment is associated with more rapid normalization of platelet counts. However, an increased risk of bleeding has also been reported, with the most common adverse events being epistaxis and gingival bleeding—most of which resolved without intervention [31]. Schofield et al. described four cases of acute thrombotic thrombocytopenic purpura (TTP) complicated by intracranial hemorrhage (ICH) in patients treated with caplacizumab. These cases were associated with poor neurological outcomes. Although refractory TTP may have contributed to the unfavorable prognoses, ICH remains a serious and recognized potential complication of caplacizumab therapy [32]. In our case, cerebellar ICH was initially detected at the local hospital. Given caplacizumab’s known risk of bleeding, its use might have exacerbated the hemorrhage, potentially increasing the risk of brainstem compression and cerebellar herniation. Furthermore, our patient’s platelet count normalized within five days following therapeutic plasma exchange alone. Therefore, the bleeding risk of caplacizumab may outweigh its potential benefits for this patient. Generally, for iTTP patients with ICH, we believe that using caplacizumab might be the most difficult decision to make and should be considered individually with extreme caution.

To the best of our knowledge, our case represents the second instance of definite iTTP complicated with DKA, and notably, it is the first to demonstrate not only clinical response but also ADAMTS-13 response. The literature contains several reports of concurrent thrombotic microangiopathy and DKA in both pediatric [11–18] and adult patients [19–21](Table 2); however, ADAMTS-13 activity below 10% was observed in only one previous case [20]. Half (5 of 12) cases had ADAMTS-13 higher than 20%, ruling out TTP, and the rest (6 of 12) had no available ADAMTS-13 results. Among all these cases, only one-third of the patients presented with platelet counts below 30 × 10^9^/L [11, 15, 17, 20], half exhibited altered consciousness [11–13, 16, 21], seizures occurred in two cases [16, 21], and a quarter required renal replacement therapy [12, 14]. Therapeutic plasma exchange (TPE) was utilized in most instances, with only two cases treated exclusively with fresh frozen plasma transfusion [17, 18]. Notably, only two cases received adjunct treatment with steroids and rituximab alongside TPE [20, 21]. Among all reported patients, only one fatality occurred [21]. From the pooled reports, DKA can occur alongside thrombotic microangiopathy (TMA), which may or may not be iTTP. Given that iTTP requires specific treatments distinct from other types of TMA, testing for ADAMTS-13 is crucial for proper clinical management. Beyond therapeutic plasma exchange (TPE), which is the standard treatment for iTTP, steroids may complicate the condition by affecting blood sugar levels and potentially exacerbating DKA. Additionally, ADAMTS-13 levels play a critical role in evaluating the remission status of iTTP. As demonstrated in this case, follow-up of ADAMTS-13 levels is equally important for guiding adjustments in immunosuppressive therapy.

**Table 2:** Case reports of concomitant thrombotic microangiopathy and diabetic ketoacidosis

	Age	Sex	Initial presentation	Lab before TPE/FFP	ADAMTS-13 activity	Treatment for TMA	Outcome
Confirmed TTP							
Jackson et al. 2021 [20]	62	male	Generalized abdominal pain, thirst, and fatigue for several days	Hb: 7.5 PLT: 18 LDH: > 2800 BUN: 78 CRE: 2.4	< 1	Steroid FFP TPE Rituximab	Improved and survived
TMA with high ADAMTS-13 activity							
Patra et al. 2011 [11]	12	female	Irritability and drowsiness for one day	Hb: 10.6 PLT: 29 LDH: 1349 BUN: 61 CRE: 2.2	52	TPE	Improved and survived
Alsaied et al. 2016 [14]	18	female	Respiratory distress few hours	Hb: 8.8 PLT: 32 LDH: >4000 CRE: 4.4	31	CRRT TPE	Improved and survived with left side weakness
Kumar et al. 2016 [15]	1	female	Vomiting for two days	Hb: 7.7 PLT: 20 LDH: 743 BUN: 67.5 CRE: 1.98	34	FFP TPE	Improved and survived
Hermelin et al. 2019 [19]	34	female	General weakness and blurred vision	Hb: 9.6 PLT: 33 LDH: 2130 CRE: 2.6	40.7	TPE	Improved and survived with blindness
Nwankwo et al. 2023 [21]	45	male	Severe abdominal pain, vomiting, and altered sensorium for one day Seizure	Hb: 8.5 PLT: 51 LDH: 588 CRE: 2.2	21	Steroid TPE Rituximab	Expired
TMA with unknown ADAMTS-13 activity							
Khan et al. 2013 [12]	14	female	Fever, vomiting, respiratory distress and LOC for one day	Hb: 7.0 PLT: 42 LDH: 1223 BUN: 75 CRE: 4.4	NA	CRRT TPE	Improved and survived
	13	female	Vomiting and LOC few hours	Hb: 8.7 PLT: 45 LDH: 1439 BUN: 39 CRE: 3.5	NA	CRRT TPE	Improved and survived
Merrick et al. 2014 [13]	9	female	LOC	PLT: 31 Deteriorating renal function	NA	TPE	Improved and survived
Mostofizadeh et al. 2018 [16]	6	female	Respiratory distress, seizure and deep coma	Hb: 8.9 PLT: 62 LDH: 1455 BUN: 60 CRE: 2.7	NA	TPE	Improved and survived
Meenakshi et al. 2020 [17]	11	male	Vomiting and abdominal pain for five days	Hb: 10.5 PLT: 16 LDH: 1179 BUN: 125 CRE: 2.7	NA	FFP	Improved and survived
Ponnambalam et al. 2020 [18]	2	male	Fever and breathlessness for 3 days	Hb: 6.2 PLT: 52 LDH: 1133 BUN: 52 CRE: 0.8	NA	FFP	Improved and survived Developmental regression (motor, language)

**Abbreviations**: TMA, thrombotic microangiopathy; TTP, thrombotic thrombocytopenic purpura; DKA, diabetic ketoacidosis; LOC, loss of consciousness; Hb, hemoglobin (g/dL); PLT, platelet count (K/µL); LDH, Lactate dehydrogenase (U/L); BUN, blood urea nitrogen (mg/dL); CRE, creatinine (mg/dL); ADAMTS-13 activity (%) (a disintegrin and metalloprotease with thrombospondin type 1 repeats, member 13); CRRT, continuous renal replacement therapy; TPE, therapeutic plasma exchange; FFP, fresh frozen plasma transfusions; NA, not available

The pathophysiologic association between TTP and diabetes mellitus is not well understood, but several mechanisms have been proposed in recent studies. Diabetes mellitus is a disease that affects the microvasculature, leading to diabetic microangiopathic changes through capillary basement membrane thickening, non-enzymatic glycosylation, increased free radical activity, and increased flux through the polyol pathway [33]. Moreover, alterations in the cholesterol-to-phospholipid ratio in the red blood cell membrane may lead to stasis within the microcirculation and increased erythrocyte mechanical fragility [34]. Hyperglycemia may also induce repression of microRNA-24 (miR-24), leading to increase von Willebrand factor (VWF) expression and secretion in diabetes mellitus [35]. Additionally, plasma ADAMTS-13 levels are decreased in diabetic nephropathy, potentially due to peripheral consumption for the cleavage of chronically increased VWF and an increase in circulating proteolytic enzymes (thrombin and plasmin) that cleave ADAMTS-13. The deterioration of renal function in diabetic nephropathy is associated with even lower ADAMTS-13 levels and a high VWF/ADAMTS-13 ratio. These mechanisms may contribute to increasing severity of iTTP and might also trigger the onset of iTTP. Therefore, the concomitant existence of TTP and DKA may not be a mere coincidence [36].

## Conclusions

This case report described a patient who presented with both DKA and iTTP simultaneously, presenting with coma and acute renal failure. Physician awareness of iTTP is crucial for prompt recognition and accurate diagnosis. Both the PLASMIC and French scores can assist in the initial identification of iTTP; however, ethnic differences and complex conditions may affect the accuracy of these prediction tools. Proposed mechanisms suggest that diabetes mellitus might exacerbate the severity of iTTP. Therefore, cases like this one, involving concomitant DKA and iTTP, may not occur purely by chance.

## Electronic supplementary material

Below is the link to the electronic supplementary material.

**Supplementary Material 1**: **Supplemental Fig.1**. Blood glucose trends and corresponding interventions throughout the clinical course

## Data Availability

No datasets were generated or analysed during the current study.

## Abbreviations

TTP: Thrombotic thrombocytopenic purpura
DKA: Diabetic ketoacidosis
ADAMTS-13: A disintegrin and metalloprotease with thrombospondin type 1 repeats, member 13
PLT: Platelet count
LDH: Lactate dehydrogenase
BUN: Blood urea nitrogen
CRE: Creatinine
CRRT: Continuous renal replacement therapy
TPE: Therapeutic plasma exchange

## References

[1] Joly BS, Coppo P, Veyradier A. Thrombotic thrombocytopenic purpura. Blood. 2017;129:2836–46. 28416507 10.1182/blood-2016-10-709857

[2] Kremer Hovinga JA, Coppo P, Lämmle B, Moake JL, Miyata T, Vanhoorelbeke K. Thrombotic thrombocytopenic purpura. Nat Rev Dis Primers. 2017;3:17020. 28382967 10.1038/nrdp.2017.20

[3] Zheng XL, Vesely SK, Cataland SR, Coppo P, Geldziler B, Iorio A, Matsumoto M, Mustafa RA, Pai M, Rock G, et al. ISTH guidelines for the diagnosis of thrombotic thrombocytopenic purpura. J Thromb Haemost. 2020;18:2486–95. 32914582 10.1111/jth.15006 PMC8146131

[4] Sukumar S, Lämmle B, Cataland SR. Thrombotic thrombocytopenic purpura: pathophysiology, diagnosis, and management. J Clin Med 2021, 10. 10.3390/jcm10030536 PMC7867179 33540569

[5] Sadler JE. Pathophysiology of thrombotic thrombocytopenic purpura. Blood. 2017;130:1181–8. 28768626 10.1182/blood-2017-04-636431 PMC5606001

[6] Zheng XL, Vesely SK, Cataland SR, Coppo P, Geldziler B, Iorio A, Matsumoto M, Mustafa RA, Pai M, Rock G, et al. ISTH guidelines for treatment of thrombotic thrombocytopenic purpura. J Thromb Haemost. 2020;18:2496–502. 32914526 10.1111/jth.15010 PMC8091490

[7] Scully M, Rayment R, Clark A, Westwood JP, Cranfield T, Gooding R, Bagot CN, Taylor A, Sankar V, Gale D, et al. A British society for haematology guideline: diagnosis and management of thrombotic thrombocytopenic purpura and thrombotic microangiopathies. Br J Haematol. 2023;203:546–63. 37586700 10.1111/bjh.19026

[8] Mariotte E, Azoulay E, Galicier L, Rondeau E, Zouiti F, Boisseau P, Poullin P, de Maistre E, Provôt F, Delmas Y, et al. Epidemiology and pathophysiology of adulthood-onset thrombotic microangiopathy with severe ADAMTS13 deficiency (thrombotic thrombocytopenic purpura): a cross-sectional analysis of the French National registry for thrombotic microangiopathy. Lancet Haematol. 2016;3:e237–245. 27132698 10.1016/S2352-3026(16)30018-7

[9] Blombery P, Kivivali L, Pepperell D, McQuilten Z, Engelbrecht S, Polizzotto MN, Phillips LE, Wood E, Cohney S. Diagnosis and management of thrombotic thrombocytopenic purpura (TTP) in Australia: findings from the first 5 years of the Australian TTP/thrombotic microangiopathy registry. Intern Med J. 2016;46:71–9. 26477687 10.1111/imj.12935

[10] Umpierrez GE, Davis GM, ElSayed NA, Fadini GP, Galindo RJ, Hirsch IB, Klonoff DC, McCoy RG, Misra S, Gabbay RA, et al. Hyperglycemic crises in adults with diabetes: A consensus report. Diabetes Care. 2024;47:1257–75. 39052901 10.2337/dci24-0032 PMC11272983

[11] Patra KP, Scott LK. Diabetic ketoacidosis preceding thrombocytopenia associated multiple organ failure in a child. Jop. 2011;12:40–3. 21206100

[12] Khan MR, Maheshwari PK, Haque A. Thrombotic microangiopathic syndrome: a novel complication of diabetic ketoacidosis. Indian Pediatr. 2013;50:697–9. 23942435 10.1007/s13312-013-0175-0

[13] Merrick V, Malik M, Vaidya M. Abstract 256: Diabetic ketoacidosis (DKA) Preceding thrombocytopenia associated with acute renal failure and pancreatic enzyme elevation. Pediatr Crit Care Med 2014, 15.

[14] Alsaied T, Goldstein SL, Kaddourah A, Poynter SE. Thrombocytopenia-associated multi-organ failure caused by diabetic ketoacidosis. Pediatr Int. 2016;58:232–4. 26712331 10.1111/ped.12780

[15] Kumar R, McSharry B, Bradbeer P, Wiltshire E, Jefferies C. Thrombocytopenia-associated multiorgan failure occurring in an infant at the onset of type 1 diabetes successfully treated with fresh frozen plasma. Clin Case Rep. 2016;4:671–4. 27386126 10.1002/ccr3.587 PMC4929803

[16] Mostofizadeh N, Arefnia S, Hashemipour M, Dehkordi EH. Thrombotic thrombocytopenic Purpura in a child with diabetic ketoacidosis. Adv Biomed Res. 2018;7:33. 29531931 10.4103/2277-9175.225928 PMC5840966

[17] Meenakshi Dadwal BKG. Parveen Kumar Antil, Baljeet Maini: thrombocytopenia associated Multi-Organ failure: A fatal complication of diabetes ketoacidosis. J Clin Diagn Res. 2020;14:SD01–2.

[18] Ponnambalam S, Varadarajan P, Subramani S, Subramanian R. Unusual complication in a child with diabetic ketoacidosis. Pediatr Oncall J. 2020;18:22–4.

[19] Hermelin D, Blackall D. Successful plasma exchange in a 34-year‐old woman with diabetic ketoacidosis and a thrombotic microangiopathy. J Clin Apheresis. 2020;35:62–5. 31652001 10.1002/jca.21751

[20] Jackson LJ, Fischer H, Abdelsayed N, Carter M. Diabetic ketoacidosis: possible cause of thrombotic thrombocytopenic purpura. Cureus 2021, 13. 10.7759/cureus.18017 PMC8520316 34667692

[21] Nwankwo CI, Samuels KA, Abung A, Oshikoya AF, Waqar D, Omole AE. Diabetic ketoacidosis complicated by thrombotic thrombocytopenic purpura: A rare association. Cureus 2023, 15. 10.7759/cureus.37983 PMC10202223 37223178

[22] Bendapudi PK, Hurwitz S, Fry A, Marques MB, Waldo SW, Li A, Sun L, Upadhyay V, Hamdan A, Brunner AM. Derivation and external validation of the PLASMIC score for rapid assessment of adults with thrombotic microangiopathies: a cohort study. Lancet Haematol. 2017;4:e157–64. 28259520 10.1016/S2352-3026(17)30026-1

[23] Coppo P, Schwarzinger M, Buffet M, Wynckel A, Clabault K, Presne C, Poullin P, Malot S, Vanhille P, Azoulay E. Predictive features of severe acquired ADAMTS13 deficiency in idiopathic thrombotic microangiopathies: the French TMA reference center experience. PLoS ONE. 2010;5:e10208. 20436664 10.1371/journal.pone.0010208 PMC2859048

[24] Li A, Khalighi PR, Wu Q, Garcia DA. External validation of the PLASMIC score: a clinical prediction tool for thrombotic thrombocytopenic purpura diagnosis and treatment. J Thromb Haemost. 2018;16:164–9. 29064619 10.1111/jth.13882 PMC5760324

[25] Lee JA, Lin MH, Kang CM, Chuang MK, Fung CKB, Lo SC. A validation and modification of PLASMIC score by adjusting the criteria of mean corpuscular volume and international normalized ratio. J Clin Apher. 2023;38:582–9. 37325919 10.1002/jca.22068

[26] Lee C-H, Huang Y-C, Li S-S, Hsu Y-T, Chen Y-P, Chen T-Y. Application of PLASMIC score in risk prediction of thrombotic thrombocytopenic purpura: Real-World experience from a tertiary medical center in Taiwan. Front Med 2022, 9. 10.3389/fmed.2022.893273 PMC9124890 35615090

[27] Tang N, Wang X, Li D, Sun Z. Validation of the PLASMIC score, a clinical prediction tool for thrombotic thrombocytopenic purpura diagnosis, in Chinese patients. Thromb Res. 2018;172:9–13. 30340093 10.1016/j.thromres.2018.10.010

[28] Matsumoto M, Bennett CL, Isonishi A, Qureshi Z, Hori Y, Hayakawa M, Yoshida Y, Yagi H, Fujimura Y. Acquired idiopathic ADAMTS13 activity deficient thrombotic thrombocytopenic purpura in a population from Japan. PLoS ONE. 2012;7:e33029. 22427934 10.1371/journal.pone.0033029 PMC3299727

[29] Tsai HM. The kidney in thrombotic thrombocytopenic purpura. Minerva Med. 2007;98:731–47. 18299685 PMC2430013

[30] Zafrani L, Mariotte E, Darmon M, Canet E, Merceron S, Boutboul D, Veyradier A, Galicier L, Azoulay E. Acute renal failure is prevalent in patients with thrombotic thrombocytopenic purpura associated with low plasma ADAMTS13 activity. J Thromb Haemost. 2015;13:380–9. 25523333 10.1111/jth.12826

[31] Scully M, Cataland SR, Peyvandi F, Coppo P, Knöbl P, Kremer Hovinga JA, Metjian A, de la Rubia J, Pavenski K, Callewaert F, et al. Caplacizumab treatment for acquired thrombotic thrombocytopenic Purpura. N Engl J Med. 2019;380:335–46. 30625070 10.1056/NEJMoa1806311

[32] Schofield J, Shaw RJ, Lester W, Thomas W, Toh CH, Dutt T. Intracranial hemorrhage in immune thrombotic thrombocytopenic purpura treated with Caplacizumab. J Thromb Haemost. 2021;19:1922–5. 33974343 10.1111/jth.15363

[33] Barnett AH. Origin of the microangiopathic changes in diabetes. Eye (Lond). 1993;7(Pt 2):218–22. 7607338 10.1038/eye.1993.52

[34] James SH, Meyers AM. Microangiopathic hemolytic Anemia as a complication of diabetes mellitus. Am J Med Sci. 1998;315:211–5. 9519937 10.1097/00000441-199803000-00013

[35] Xiang Y, Cheng J, Wang D, Hu X, Xie Y, Stitham J, Atteya G, Du J, Tang WH, Lee SH, et al. Hyperglycemia repression of miR-24 coordinately upregulates endothelial cell expression and secretion of von Willebrand factor. Blood. 2015;125:3377–87. 25814526 10.1182/blood-2015-01-620278 PMC4447857

[36] Taniguchi S, Hashiguchi T, Ono T, Takenouchi K, Nakayama K, Kawano T, Kato K, Matsushita R, Nagatomo M, Nakamura S, et al. Association between reduced ADAMTS13 and diabetic nephropathy. Thromb Res. 2010;125:e310–316. 20307901 10.1016/j.thromres.2010.02.013

