# Supplementary figures and images for: Immune thrombotic thrombocytopenic purpura and diabetic ketoacidosis: a case report and literature review
Source: Thromb J. 2025 May 19;23:50. doi: 10.1186/s12959-025-00740-w (PMC12087192; doi:10.1186/s12959-025-00740-w)

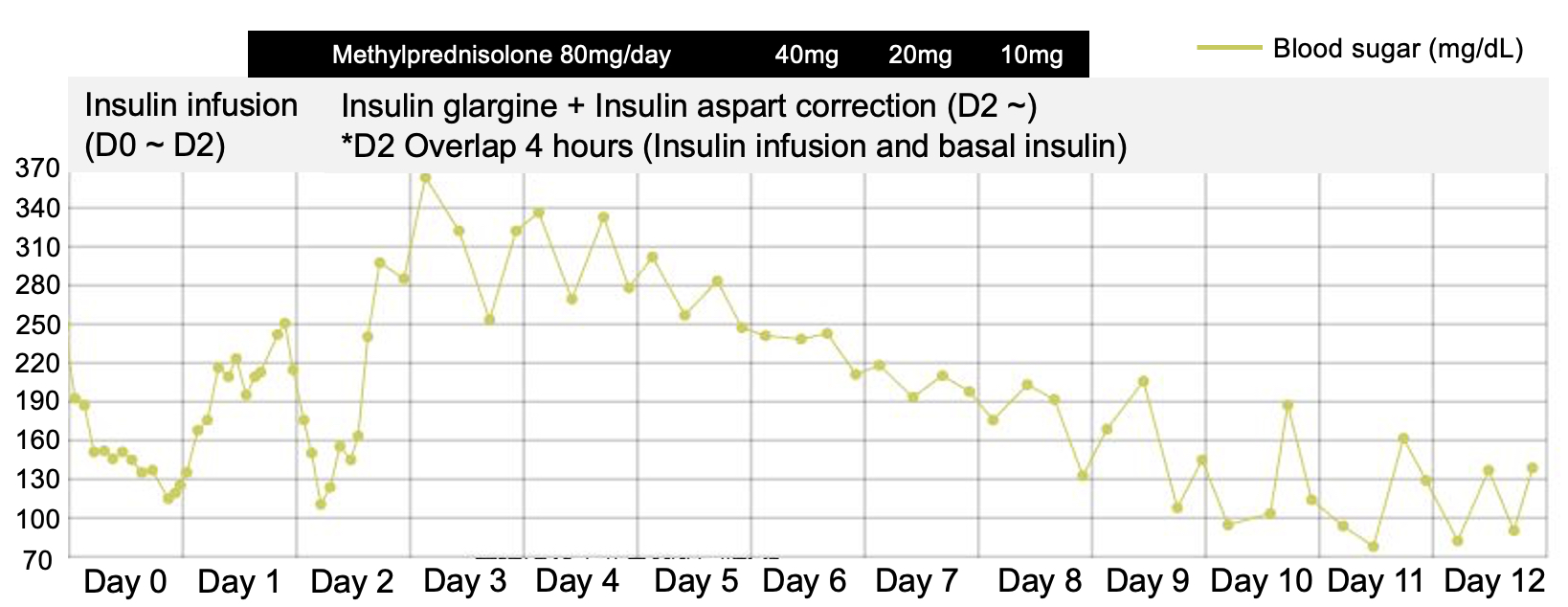

Supplement: Supplementary file 1 — Supplementary Material 1: Supplemental Fig.1. Blood glucose trends and corresponding interventions throughout the clinical course [file 12959_2025_740_MOESM1_ESM.jpg]
